# Incidence of Hyperuricemia in Patients with Acute Myocardial Infarction – A Case-Control Study

**DOI:** 10.7759/cureus.6722

**Published:** 2020-01-21

**Authors:** Kheraj Mal, Jamila Begum Jabar Ali, Kiran Fatima, Amber Rizwan

**Affiliations:** 1 Cardiology, National Institute of Cardiovascular Diseases, Sukkur, PAK; 2 Internal Medicine, Rajiv Gandhi University of Health Sciences, Chitradurga, IND; 3 Internal Medicine, Jinnah Post Graduate Medical Center, Karachi, PAK; 4 Family Medicine, Dr. Ruth Pfau Hospital, Karachi, PAK

**Keywords:** hyperuricemia, myocardial infarction, incidence, case control, pakistan

## Abstract

Introduction

Various researches have stated the correlation between serum uric acid (sUA) and cardiovascular disease (CVD); however, no local studies are available. In this study, we will determine the prevalence of hyperuricemia in patients with acute myocardial infarction and compare with the control group.

Methods

This case-control study was conducted from March to November 2019 in the tertiary care hospital in Pakistan. In all, 119 patients with acute myocardial infarction were enrolled in this study, and 119 controls were identified from the outpatient department. Their sUA levels were measured within 24 hours of acute myocardial infarction.

Results

The mean sUA levels were significantly higher in patients with acute myocardial infarction (AMI) in comparison to the control group (6.17 ± 2.12 vs. 5.51 ± 1.89, *p*-value; 0.01). Overall, there were more patients with hyperuricemia in the case group compared to the control group (47.89% vs. 33.6%, *p*-value = 0.04)

Conclusion

In this study, after adjustment of other known factors, hyperuricemia is associated with AMI. Efforts should be made to include screening for hyperuricemia in patients with a high risk of myocardial infarction.

## Introduction

Pakistan has a high prevalence of hyperuricemia [1]. Although there is no large-scale epidemiological report, several smaller studies in this decade have reported a frequency of 30% to 39% [1-2]. Hyperuricemia most commonly results in gouty arthritis, which presents as tender, swollen, inflamed joints due to crystal deposition. Gouty arthritis is more commonly seen in men [3].

Over time, researchers have linked gouty arthritis to cardiovascular comorbidity. Large epidemiological data have established a strong association of cardiovascular disease (CVD) with gouty arthritis [4-6]. Other studies have also established hyperuricemia, with or without gout, as a precursor and independent risk factor for a range of CVDs including hypertension (HTN), metabolic syndrome, and coronary artery disease (CAD) [7-10]. In a recent study with 2000+ patients, Ranjith et al. determined that increased serum uric acid (sUA) was not only significantly with associated with HTN and renal dysfunction but also major adverse cardiac events (MACE). They also concluded that acute myocardial infarction (AMI) patients with hyperuricemia had an increased risk of mortality [11]. The possible explanation for an increased risk of CVD in patients with hyperuricemia can be the role of uric acid in HTN, oxidative stress, endothelial dysfunction and metabolic syndrome [11].

As discussed above, the frequency of hyperuricemia in Pakistan is 30% to 39% and the frequency of CVDs is 17.5% [2-3,12]. However, to the best of our knowledge, no study has been conducted to evaluate the correlation between the two in our population. Hence, in this study, we aimed to assess sUA levels in patients with AMI and compare them with healthy adults.

## Materials and methods

We conducted a case-control study in a tertiary public hospital in Pakistan. This study was conducted from 1st March to 30th November, 2019. All patients were included after attaining informed consent. The study was approved by the institutional review board.

Patients who were admitted with AMI within the last 24 hours, confirmed by history, electrocardiogram (ECG), and cardiac biomarkers were included as “cases”. Their healthy counterparts were randomly recruited from the attendants of the patients as “controls”. For both groups, demographic and clinical characters including gender, age, smoking status, comorbidities, previous history of myocardial infarction, family cardiac history, and use of thiazide were recorded. Peripheral venous blood samples were collected within 24 h to determine sUA levels from the in-hospital laboratory facility. Hyperuricemia was defined as sUA levels greater than 5.8 mg/dl in women and 7 mg/dl in men [13].

Statistical analysis was done using SPSS v. 22.0 (IBM Corp., Armonk, NY, US). Continuous variables were analyzed via descriptive statistics and were presented as mean and standard deviation (SD) while categorical variables were presented by percentages and frequencies. An independent sample T-test was applied to compare continuous variables. Chi-square was applied to compare categorical variables. P-value ≤0.05 was taken as significant.

## Results

This study was completed by 119 cases and 119 controls. Their demographic characteristics such as age, smoking status, co-morbidities such as smoking, diabetes, hypertension, and other risk factors for AMI were comparable between the two groups. Mean uric acid levels were significantly higher in patients with AMI in comparison to the control group (Table 1).

**Table 1:** Comparison of demographic and clinical characteristics of the cases and the controls AMI, acute myocardial infarction

Patient characteristics	Cases (n = 119)	Controls (n = 119)	P-value
Age in years	58 ± 17	55 ± 19	0.20
Gender			
Male	71 (59.66%)	66 (55.46%)	0.43
Female	48 (40.33%)	53 (44.53%)	
Active smoking	51 (42.85%)	53 (44.53%)	0.79
Diabetes mellitus	31 (26.05%)	25 (21.00%)	0.84
Hypertension	61 (51.26%)	56 (47.05%)	0.51
Use of Thiazides	31 (26.05%)	27 (22.68%)	0.54
Previous history of AMI	9 (7.56%)	6 (5.04%)	0.42
Family history of AMI	12 (10.08%)	11 (9.24%)	0.82
Mean serum uric acid in mg/dl	6.17 ± 2.12	5.51 ± 1.89	0.01

The sUA level was then categorized according to patient gender. In both cases and controls, men had a higher frequency of hyperuricemia than women. Men in the cases group had a higher frequency of hyperuricemia than men in the controls (53% vs. 36%). The differences were statistically significant (*p *= 0.04). The frequency of hyperuricemia in women of both groups did not differ statistically. Overall, cases had a higher frequency of hyperuricemia than controls (48% vs. 33%). The differences were statistically significant (*p *= 0.04). All data are summarized in Table 2.

**Table 2:** Frequency of hyperuricemia deficiency in the cases and the controls

Gender	Frequency of hyperuricemia		P-value
	Cases (n = 119)	Controls (n = 119)	
Male	38 (53.52%)	24 (36.36%)	0.04
Female	19 (39.58%)	16 (30.1%)	0.32
Total	57 (47.89%)	40 (33.6%)	0.04

## Discussion

Hyperuricemia has been correlated with various diseases such as hypertension, hyperlipidemia, diabetes, metabolic syndrome, and renal disease, which all have contributed to increased coronary heart disease (CHD) and all-cause mortality. In this study, mean uric acid was higher in patients with AMI compared to the control group. In addition, there were more patients with hyperuricemia in case group compared to control group. There were fifty-seven (47.89%) participants with hyperuricemia in patients. The control population had 33.6% participants with hyperuricemia. This was comparable to hyperuicemia reported in other local literature [1]. In our study, the association was significant for men as compared to women. This was in contrast to the results of a meta-analysis where hyperuricemia was associated with the risk of CAD-related mortality; however, the association was stronger for women than men [14].

There have been various explanations to the association of high sUA with CVDs. One model postulates that uric acid is one of the risk factors for endothelial dysfunction, which plays a major role in cardiovascular diseases. Uric acid increases high mobility group box chromosomal protein 1 (HMGB1) expression and extracellular release in endothelial cells. HMGB1, which is an inflammatory cytokine, interacts with the Receptor for Advanced Glycation products (RAGE) which leads to oxidative stress and inflammatory response, which causes endothelial dysfunction [15]. RAGE has been associated with atherosclerosis as it is expressed on various cells such as due to its expression on the surface of a wide variety of cells, such as endothelial cells, lymphocytes, monocyte-derived macrophages, and vascular smooth muscle cells, that are responsible for the pathogenesis of atherosclerosis [16]. Other studies have also studied the role of uric acid in endothelial dysfunction [17-18].

Among our AMI patients, 57 (47.89%) had hyperuricemia as compared to 40 (33.6%) controls. Mean uric acid levels were also higher in AMI patients. In an Indian study, the sUA levels were higher in patients of AMI correlated with Killip class [19]. Li-chen established that not only higher sUA is associated with increased myocardial infarction incidence but also patients with higher uric acid had a higher rate of left systolic dysfunction and diastolic dysfunction and were likely to have more in-hospital MACEs [20].

The study has its limitations. First, since it was a case-control study, a strong correlation could not be established. A prospective cohort study is needed to further establish uric acid as the risk factor for AMI in the Pakistani population. Second, since it was a single-center study, the result could not be inferred to a larger population.

## Conclusions

In this study, uric acid levels were higher in patients with AMI and there were more patients with hyperuricemia compared to the control group. Uric acid should be considered as a strong risk factor for AMI and should be included in general screening by cardiologists and health-care professionals. Furthermore, a large-scale prospective cohort study is needed to establish the correlation between uric acid and AMI.

## References

[1] Qudwai W, Jawaid M (2017). Frequency of uric acid levels symptomatic and asymptomatic hyperuricemia among the Pakistani population. Mid East J Fam Med.

[2] Raja S, Kumar A, Aahooja R D (2019). Frequency of hyperuricemia and its risk factors in the adult population. Cureus.

[3] Jin M, Yang F, Yang I, Yin Y, Luo JJ, Wang H, Yang XF (2012). Uric acid, hyperuricemia and vascular diseases. Front Biosci.

[4] Abbott RD, Brand FN, Kannel WB, Castelli WP (1988). Gout and coronary heart disease: the Framingham Study. J Clin Epidemiol.

[5] Krishnan E, Svendsen K, Neaton JD, Grandits G, Kuller LH (2008). Long-term cardiovascular mortality among middle-aged men with gout. Arch Intern Med.

[6] Krishnan E, Baker JF, Furst DE, Schumacher HR (2006). Gout and the risk of acute myocardial infarction. Arthritis Rheum.

[7] Schretlen DJ, Inscore AB, Vannorsdall TD, Kraut M, Pearlson GD, Gordon B, Jinnah HA (2007). Serum uric acid and brain ischemia in normal elderly adults. Neurology.

[8] Roberts JM, Bodnar LM, Lain KY, Hubel CA, Markovic N, Ness RB, Powers RW (2005). Uric acid is as important as proteinuria in identifying fetal risk in women with gestational hypertension. Hypertension.

[9] Ford ES, Li C, Cook S, Choi HK (2007). Serum concentrations of uric acid and the metabolic syndrome among US children and adolescents. Circulation.

[10] Tuttle KR, Short RA, Johnson RJ (2001). Sex differences in uric acid and risk factors for coronary artery disease. Am J Cardiol.

[11] Ranjith N, Myeni NN, Sartorius B (2017). Association between hyperuricemia and major adverse cardiac events in patients with acute myocardial infarction. Metab Syndr Relat Disord.

[12] Zubair F, Nawaz SK, Nawaz A, Nangyal H, Amjad N, Khan MS (2018). Prevalence of cardiovascular diseases in Punjab, Pakistan: a cross-sectional study. J Public Health.

[13] Williamson MA, Snyder LM (2014). Wallach's Interpretation of Diagnostic Tests: Pathways to Arriving at a Clinical Diagnosis. Wolters Ktuwer.

[14] Kim YS, Guevara JP, Kim KM, Choi HK, Heitjan DF, Albert DA (2010). Hyperuricemia and coronary heart disease: A systematic review and meta-analysis. Arthritis Care Res.

[15] Cai W, Duan XM, Liu Y (2017). Uric acid induces endothelial dysfunction by activating the HMGB1/RAGE signaling pathway. Biomed Res Int.

[16] Goldin A, Beckman JA, Schmidt AM, Creager MA (2006). Advanced glycation end products: sparking the development of diabetic vascular injury. Circulation.

[17] Feig DI, Johnson RJ (2007). The role of uric acid in pediatric hypertension. J Renal Nutr.

[18] Feig DI, Kang DH, Johnson RJ (2008). Uric acid and cardiovascular risk. New Engl J Med.

[19] Nadkar MY, Jain VI (2008). Serum uric acid in acute myocardial infarction. J Assoc Physicians.

[20] Chen L, Li XL, Qiao W (2012). Serum uric acid in patients with acute ST-elevation myocardial infarction. World J Emerg Med.

